# Ethical, Legal, and Social Issues Related to the Inclusion of Individuals With Intellectual Disabilities in Electronic Health Record Research: Scoping Review

**DOI:** 10.2196/16734

**Published:** 2020-05-21

**Authors:** Melissa Raspa, Rebecca Moultrie, Laura Wagner, Anne Edwards, Sara Andrews, Mary Katherine Frisch, Lauren Turner-Brown, Anne Wheeler

**Affiliations:** 1 RTI International Research Triangle Park, NC United States; 2 The University of North Carolina at Chapel Hill TEACCH Autism Program Chapel Hill, NC United States

**Keywords:** electronic health records, privacy, informed consent, intellectual disability, genetics

## Abstract

**Background:**

Data from electronic health records (EHRs) are increasingly used in the field of genetic research to further precision medicine initiatives. However, many of these efforts exclude individuals with intellectual disabilities, which often stem from genetic conditions. To include this important subpopulation in EHR research, important ethical, legal, and social issues should be considered.

**Objective:**

The goal of this study was to review prior research to better understand what ethical, legal, and social issues may need further investigation when considering the research use of EHRs for individuals with genetic conditions that may result in intellectual disability. This information will be valuable in developing methods and best practices for involving this group in research given they are considered a vulnerable population that may need special research protections.

**Methods:**

We conducted a scoping review to examine issues related to the use of EHRs for research purposes and those more broadly associated with genetic research. The initial search yielded a total of 460 unique citations. We used an evaluative coding process to determine relevancy for inclusion.

**Results:**

This approach resulted in 59 articles in the following areas: informed consent, privacy and security, return of results, and vulnerable populations. The review included several models of garnering informed consent in EHR or genetic research, including tiered or categorical, blanket or general, open, and opt-out models. Second, studies reported on patients’ concerns regarding the privacy and security of EHR or genetic data, such as who has access, type of data use in research, identifiability, and risks associated with privacy breach. The literature on return of research results using biospecimens examined the dissension in the field, particularly when sharing individualized genetic results. Finally, work involving vulnerable populations highlighted special considerations when conducting EHR or genetic research.

**Conclusions:**

The results frame important questions for researchers to consider when designing EHR studies, which include individuals with intellectual disabilities, including appropriate safeguards and protections.

## Introduction

### Background

The field of genetics has grown exponentially over the last decade. Advances in whole genome and exome sequencing have made the diagnosis of genetic conditions easier than ever before. Conditions diagnosed through these approaches run the gamut from cancer to rare diseases [1-4]. Genetic testing also is increasingly being integrated into clinical care and viewed as a common practice to detect genetic causes of conditions [5]. Another mechanism for genetic discovery lies with the increasing use of electronic health records (EHRs). EHRs have become a more commonplace tool in genetics, allowing researchers to conduct studies on specific genetic conditions, including observational, epidemiological, descriptive, and comparative effectiveness studies, among others [6,7]. EHRs are also being used to recruit participants for research studies and clinical trials [8].

Adding to their utility, EHRs can be linked with biobank data to answer genotype and phenotype research questions [9]. For example, the Electronic Medical Records and Genomics (eMERGE) network, which began in 2007, uses biorepositories linked to EHRs to conduct electronic phenotyping to identify patterns and diagnose disease [10-12]. More recently, the movement toward precision medicine has inspired other similar initiatives, such as Geisinger’s MyCode Community Health Initiative [13] and *All of Us*, sponsored by the National Institutes of Health [14]. Other work has focused on phenome-wide association studies that analyze different phenotypes to determine a genetic variant [15].

All of these programs are designed with the promise of providing tailored health interventions based on an individual’s genetic makeup. Ultimately, this may lead to a reduction in health disparities but only if a diverse array of individuals is included [16]. With this in mind, there have been calls to expand enrollment to underrepresented individuals, including those with intellectual disabilities [17,18]. Given that many conditions that results in intellectual disability can have genetic underpinnings, the inclusion of this subgroup in EHR research can provide insights into biological causes to various diseases and comorbidities [17].

### Objectives

Previous work has examined ethical, legal, and social issues related to EHR use as well as combining EHRs with genetic data. However, research conducted to date has focused on typically developing individuals (ie, individuals with no known or suspected genetic conditions) who wanted to contribute their data to advance science and clinical practice [19], and thus, has predominantly excluded individuals with specific genetic conditions or those with intellectual disabilities. The inclusion of this group of individuals achieves the goal of making research, in particular the precision medicine initiative, more representative and patient-centered, and also aligns with the desires of those with disabilities [20]. Thus, there is a need to further explore the barriers or challenges of including this group of individuals in EHR research.

The goal of this study was to review existing studies to better understand what ethical, legal, and social issues may need further investigation when considering the research use of EHRs for individuals with genetic conditions that may result in intellectual disability. This information will be valuable in developing methods and best practices for involving this group in future research.

## Methods

### Design

We conducted a scoping review of the literature to examine ethical, legal, and social issues related to the use of EHRs for research purposes or issues more broadly associated with genetic research. We chose a scoping review approach given the breadth of literature in this area. This also enabled us to synthesize information from diverse sources, including theoretical and narrative reviews, qualitative studies, and quantitative research. We first identified our search terms; conducted a search of relevant literature; reviewed, charted, and collated the information; and summarized key findings [21,22].

### Search Terms

Given that we were interested in ethical, legal, and social issues related to the inclusion of individuals with genetic conditions that resulted in intellectual disability in EHR research, we conducted searches for the following two topic areas: (1) research use of EHRs and (2) ethical, legal, and social issues of genetics research. The research team developed search terms for each topic area **(**Textbox 1**)**. Inclusion criteria included peer review articles published in English between 2000 and 2018. We conducted our search using the following databases: PubMed (Medical Literature Analysis and Retrieval System Online, MEDLINE), Cumulative Index to Nursing and Allied Health Literature (CINAHL), Cochrane Review, PsycINFO, and Web of Science.

**Search terms by topic area.**

Research use of electronic health records

- medical record, health record, electronic record, electronic health information, health data, clinical record, clinical data, Health Insurance Portability and Accountability Act, HIPAA, PHR, EMR, EHR, eHealth, or e-Health, and
- outcome, research, measur*, assess*, evaluat*, analy*, study, or studies, and
- cognitive decline, cognitive impairment*, intellectual disabilit*, developmental disabilit*, autism, fragile X, Alzheimer*, genetic*, genomic*, vulnerable population, cognitively impaired, intellectually disabled, developmentally disabled, autistic,

Ethical, legal, and social implications of genetics research

- ethical issue, legal issue, social issue, ethical implication, ethical requirement, guardian, ethic, or social, and
- consent issue, informed consent, consent, medical record, health record, electronic record, electronic medical record, electronic health record, electronic health information, health data, clinical record, clinical data, Health Insurance Portability and Accountability Act, HIPAA, PHR, EMR, EHR, eHealth, or e-Health, and
- cognitive decline, cognitive impairment, intellectual disabilit*, developmental disabilit*, autism, fragile X, Alzheimer*, genetic*, genomic*, cognitively impaired, intellectually disabled, developmentally disabled, autistic

### Abstract Review

**Figure 1:**
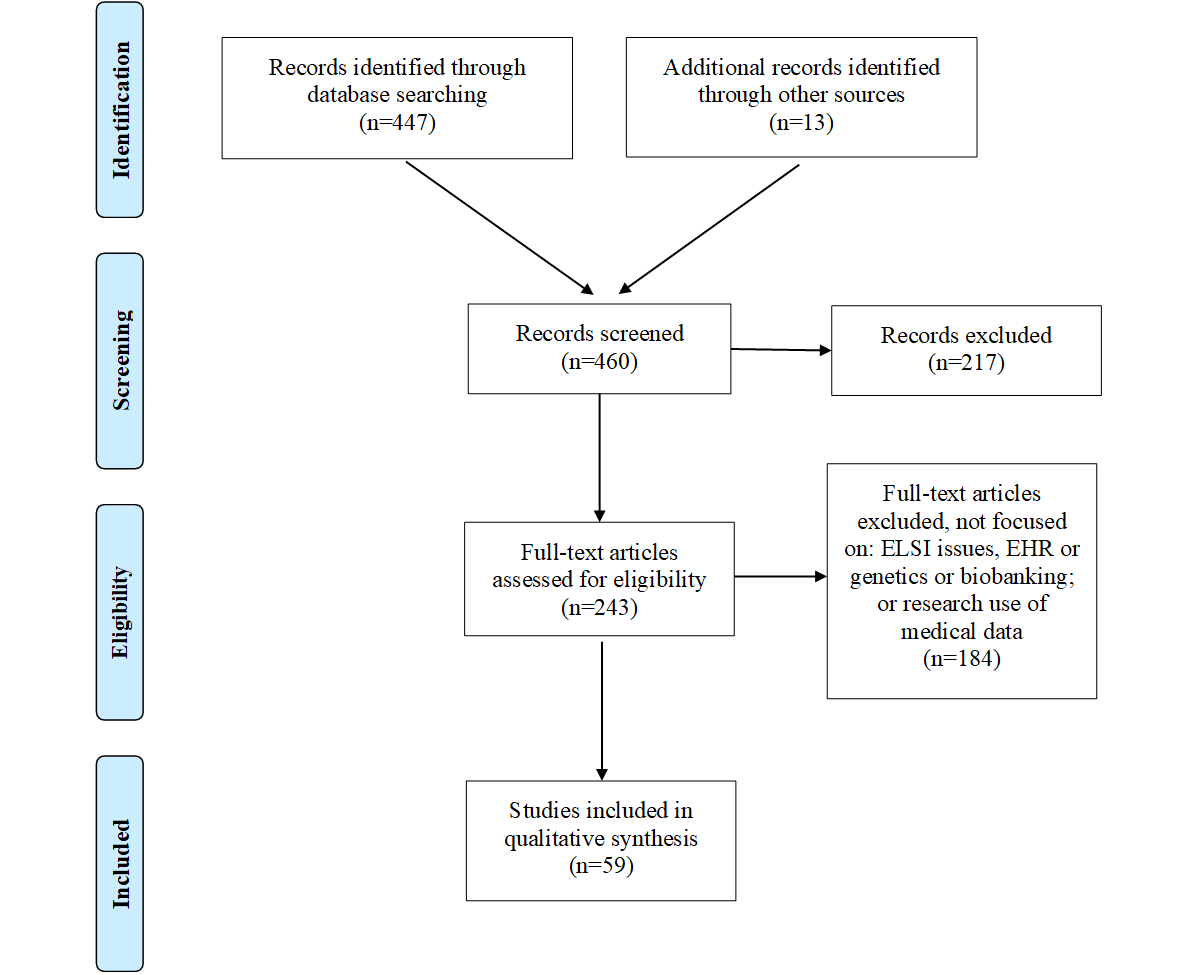
Preferred Reporting Items for Systematic Reviews and Meta-Analyses flowchart. EHR: electronic health record; ELSI: ethical, legal, and social issues.

The initial search yielded a total of 447 unique citations (duplicates removed): 206 in the research use of EHRs topic area and 241 in the ethical, legal, and social issues of genetics research topic area. We identified an additional 13 articles outside our initial search parameters, mainly from ethics journals that were not indexed in MEDLINE. The study team developed a coding structure in which each article was scored on a 4-point scale, with 0 being not at all relevant and 3 being extremely relevant. A pair of researchers were assigned the same abstracts and conducted blind coding of each to determine relevance for inclusion and whether a full-text review of the article was appropriate. The pair met to review and compare scores. In cases where scores did not match, researchers discussed and arrived at a consensus score. In total, 243 abstracts were deemed relevant (scored a 2 or 3) to obtain the full text. This included 120 in the research use of EHRs category and 123 in the ethical, legal, and social implications of genetics research category. The full-text articles were then reviewed, key findings were extracted and charted, and themes were categorized. The scoping review culminated in a total of 62 articles: 26 on the research use of EHRs and 36 on the ethical, legal, and social issues of genetics research. These full-text articles were reviewed, and themes were extracted (see Figure 1).

## Results

### Overview

**Figure 2:**
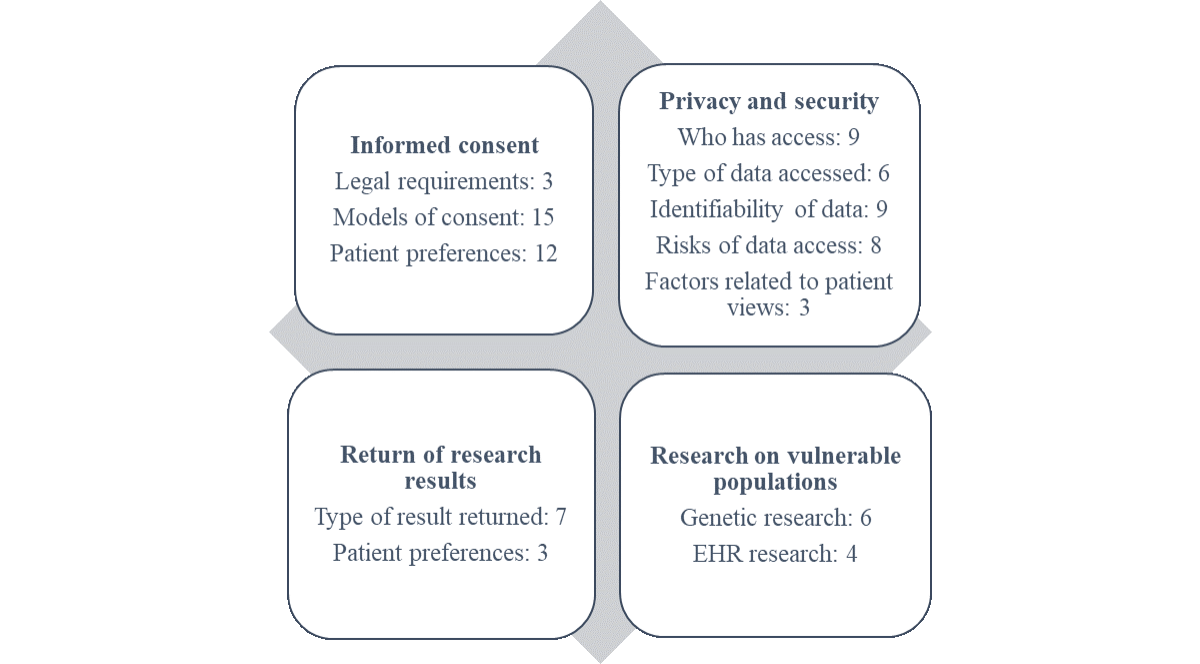
Number of articles included by ethical, legal, and social issue. EHR: electronic health record.

We grouped the results into four broad ethical, legal, and social issues related to EHRs and genetic research that would applicable to those with intellectual disabilities (see Figure 2). The first section covers issues related to informed consent for the use of EHRs in research. This section includes a review of the legal requirements in the United States and also describes possible models of obtaining informed consent for EHRs and genetics research. The next section provides an overview of issues related to the privacy and security of EHRs and genetics research, including studies that examined preferences for who has access to information in the health record and what information is accessed. The third section examines the return of research results, which is most often considered within the context of genetics and biobank research. The final section discusses unique considerations for conducting research with vulnerable populations.

### Informed Consent

#### Legal Requirements of Informed Consent

The first issue faced when conducting research with EHRs or on genetic conditions is whether informed consent is needed [23,24]. As set forth in the Common Rule and specified in US regulatory requirements, anyone conducting research with human subjects must have an institutional review board (IRB) review their study before initiation. Under the new federal regulations, referred to as the Final Rule [25], the definition of research remains unchanged as “a systematic investigation, including research development, testing, and evaluation, designed to develop or contribute to generalizable knowledge” (see 45CFR46.102(l)). However, the classification of “human subjects” now includes detailed definitions about identifiable private information (ie, “private information for which the identity of the subject is or may readily be ascertained by the investigator or associated with the information”) and identifiable biospecimens (ie, “a biospecimen for which the identity of the subject is or may readily be ascertained by the investigator or associated with the biospecimen;” see 45CFR46.102(e)).

If informed consent is required, it can be obtained directly from the research participant or the participant’s legally authorized representative. A legally authorized representative is determined by law or court authority. Under the Final Rule, individuals with intellectual disabilities or those with genetic conditions are not specifically referenced. However, research participants who may be “vulnerable to coercion or undue influence” or those with “impaired decision-making ability” are mentioned. Examples of these potential participants may include children, prisoners, or those with intellectual disabilities.

In the informed consent form, researchers need to include whether any identifiable private information will be used as part of the study. It is unclear if a specific genetic condition, intellectual disability would fall under the definition of private identifiable information within the Final Rule. The code simply mentions that definition of will be reviewed regularly and updated as needed. Until then it appears that agencies who implement this policy will need to define what falls under the umbrella of identifiable private information. The Health Insurance Portability and Accountability Act (HIPAA) Privacy Rule (see 45CFR160-164), which covers EHRs, does define genetic information and considers it protected health information. Like the Final Rule, HIPAA has specific requirements about sharing information, including data from an individual’s EHR. Thus, EHR research on specific genetic conditions would need to be reviewed by an IRB to determine whether informed consent should be obtained from participants.

There are cases, however, when a waiver of informed consent can be granted. The following criteria must be met to request a waiver: (1) the research involves no more than minimal risk to the subjects; (2) the research could not practicably be carried out without the requested waiver or alteration; (3) if the research involves using identifiable private information or identifiable biospecimens, the research could not practicably be carried out without using such information or biospecimens in an identifiable format; (4) the waiver or alteration will not adversely affect the rights and welfare of the subjects; and (5) whenever appropriate, the subjects or legally authorized representatives will be provided with additional pertinent information after participation (see 45CFR46.116(f)). In general, local IRBs need to interpret the regulations and determine if a study qualifies as research on human subjects, and if so, whether a waiver of consent can be granted when conducting EHR research on genetic conditions.

When researchers meet all the requirements of a waiver of informed consent, it is often under the auspices of conducting research for the common good [26]. Examples include research aimed at improving public health or the quality of medical care. However, EHR research without consent may violate the ethical principles of autonomy in the hopes of achieving beneficence and justice [27]. However, many bioethicists have argued in favor of EHR research without consent as long as the aforementioned requirements of a waiver are met, and proper privacy and security restrictions are in place [26,28,29].

#### Models of Informed Consent

If informed consent is not waived, several different models have been proposed for meeting human subject protection requirements and minimizing researcher burden. A tiered, or categorical, consent model that offers research participants options for how and when their data can be used has been espoused as best practice in the field on genetics research [30,31]. A tiered consent model allows participants to choose from many options, such as allowing their data to be used for the current study only, the current study as well as future studies about a specific condition or type of research, or unlimited use of their data [23,32,33]. This type of consent model could also include an option for reconsent, such as when a new subcategory of a genotype or phenotype for a particular condition is discovered that may need an additional level of consent before further research is conducted [34]. Although this approach is in line with the Belmont principle of *respect for persons* and encourages autonomy among research participants, some have criticized it given that it may be logistically challenging to implement and could potentially diminish the utility and applicability of clinical data, whether information in EHRs or biospecimens, for future research purposes [35,36]. A recent evolution of the tiered consent model is dynamic consent in which researchers and participants have ongoing communication that allows participants to update their consent preferences over time and enables researchers to return results to those interested [37].

A second approach to informed consent is the use of a blanket, or general, consent model [12,23,34]. Blanket consent can be described as an all-or-nothing option for using EHR data or biospecimens in future research. In other words, participants are given the choice to consent to all future research use of their data without being given the details of what that research may entail. The National Institutes of Health now asks funded investigators to include broad data sharing as part of their informed consent forms for genetic research studies [38]. Similar to the tiered consent model, there are advocates and critics of this approach. Proponents cite that it covers all the necessary ethical and legal requirements of informed consent, whereas detractors argue that the lack of specifics on future use means that it is more akin to providing permission than consent that is truly informed [39,40].

The open consent model, used by the Personal Genome Project, takes the blanket consent model a step further by stating up front that participants’ data may be accessed broadly and there are no guarantees of anonymity, privacy, or confidentiality [34]. The goal of this approach is to provide more specifics about the possible ways data may be used and the risks of participating. Advocates of this approach argue that being open and honest about the study design and potential uses of data will promote trust and willingness to participate.

A final model of informed consent that has been proposed involves opting out of research participation. The opt-out model requires a participant to actively withdraw from the research. If they do not withdraw, it is assumed they passively consent to participate. This model was adopted by the Iceland biobank project, a national effort to merge three sources of data—EHR, genealogical, and genetic—into one database for research purposes [23]. The opt-out model, however, was not consistently applied across the data sources and was met with criticism by those who felt it did not offer adequate control for research participants or meet ethical and legal requirements [32]. It seems there is no uniform approach to seeking consent for biobank research [41].

Few studies have focused on informed consent models for accessing only EHR data, without a link to biospecimens. In a commentary on whether it is ethical to access EHR data, even identifiable information, without consent, Miller asserts the answer lies in the balance between risks and benefits [26]. In cases where the public good outweighs the personal risks to an individual, then informed consent can be waived under the auspices of a utilitarian philosophy. However, the objection to this approach is that it violates an individual’s right to privacy.

Ultimately, under the Final Rule and HIPAA Privacy Rule, this decision rests in the hands of IRBs. However, in a study of IRB chairs and administrators that were presented with a hypothetical EHR research protocol, opinions varied widely as to whether informed consent was needed, even when identifying information was not included [42]. IRB chairs and administrators who stated that consent was needed reasoned that there was a risk for reidentification. Even though identifiable information (eg, last name) was not being extracted, other information such as birth date, postal code, and ethnic origin were included. IRB chairs and administrators who said consent was not needed stated that the data were anonymous or that the identifiability of participants was not relevant to determining if consent was required.

#### Patient Preferences Related to Informed Consent

Several US-based studies have examined the preferences of patients related to providing informed consent for use of clinical data, including both biospecimens and EHRs. In one large survey study, most patients were willing to participate in a biobank [43]. However, some studies suggest that participants tend to prefer a broad consent model, as long as no personally identifiable information is used [44,45]. In another study, public sharing of deidentified data was favored by the majority of participants when either a tiered or blanket option was given [46]. Sharing of data has not been shown to be related to level of understanding or recall of the informed consent process [47]. However, preferences for models of consent have been shown to vary by some sociodemographic factors, such as race, ethnicity, income, and education [43,45,46].

Consent preferences are also driven by what information is being accessed and how it is being used. Patients favored seeking consent for the use of medical history data and treatment-related information [48]. Reconsent was preferred when researchers were investigating a health condition that was unrelated to the one for which they originally gave consent, if deidentified data were shared with an investigator at a different institution, and when a child for whom a parent had previously given consent had reached adulthood [43,49]. Patients’ decision to consent to research use of their biospecimen is also related to personal characteristics, including whether a participant has an existing genetic condition [49].

Studies in Europe and Canada have found similar preferences. Surveys and focus groups with patients in Ireland and the United Kingdom indicated a preference for some level of control over use of EHR data for research, with many favoring a study-by-study or tiered consent model [50-52]. In an Italian study, over half of those enrolled in a national twin registry were against the use of their EHR data without informed consent [53]. Similarly, in Canada, participants preferred to be asked for consent either verbally or in writing before releasing their EHR data to researchers [54].

### Privacy and Security

#### Who Has Access to Data for Research Use

One of the most common ethical, legal, and social issues related to using EHRs for research is who has access [27,33,55]. Patients often had the most concern about health insurance and pharmaceutical companies as well as government agencies using their EHR data for research purposes [44,54,56-58]. Pereira et al [59] found that 66.9% (328/490), 44.9% (220/490), and 40.0% (196/490), respectively, did not want insurance companies, pharmaceutical companies, and the government to have access to their health information. Patients worried that research conducted by pharmaceutical companies would be used to promote products, and there was potential for insurance companies to deny coverage for patients [54]. These companies were seen as less “legitimate” to patients because the research they were likely to conduct was not “pure science,” which aimed to generate knowledge for the common good [58]. Patients were also concerned with unauthorized access to their medical information or sharing of EHR data with noncredible researchers [57]. One example cited was government researchers accessing EHR data but then selling it to a pharmaceutical company [56]. Therefore, patients often desired greater oversight of individuals who conducted EHR research within these organizations [44]. Typically, the amount of trust a patient had in the researcher or organization conducting the research was related to the patient’s views on privacy [44,51,54,56].

#### What Type of Data Are Accessed for Research

Some patients reported that they would prefer to limit access to certain information in their medical records to maintain privacy [58,60]. Patients were less likely to share sensitive information such as substance abuse history, mental health information, sexual health information, domestic violence records, reproductive health records, and genetic information and would want to be asked permission before sharing this information with researchers [60]. In particular, patients were concerned with the disclosure of conditions viewed as stigmatizing, such as HIV/AIDS and mental illness, to researchers [56-58]. One study reported that patients viewed reproductive and mental health information as more sensitive than genetic data [58]. To ensure privacy, some patients wanted to limit access to portions of their EHR to their health care provider [61] or not include any sensitive information in their EHR [16,33].

#### Identifiability of Data

Despite widespread support for research use of EHR data and biospecimens, patients often preferred that their information remain anonymous [49,51]. Hull [62] conducted interviews with patients of academic medical centers to examine attitudes and preferences on the use of anonymous versus identifiable samples for genetic research. On the basis of responses to two hypothetical scenarios, 72.96% (850/1165) preferred to be informed about the use of their anonymous blood samples, whereas 83.35% (971/1165) of patients wanted to be told if it was an identifiable sample. However, only 23.38% (271/1159) of patients responded differently to the two scenarios, with most (196/1159, 16.91%) indicating they wanted to be notified about the use of an identifiable sample and did not need to know if it was done with an anonymous sample.

In biobanking research, similar privacy concerns have been raised about lack of oversight and the potential for biospecimens to be identified [63,64]. Although technology has enabled large-scale genomic studies to be conducted, the sharing of data with outside researchers and linking of biospecimens with EHRs has increased the potential for reidentification of participants [23,63]. Improved data mining technology has also contributed to privacy and identifiability concerns [65]. Despite the fact that some research has shown that reidentification of biospecimens and EHR data is difficult when proper security controls are in place [66,67], concerns about privacy and security are likely to be an ongoing issue.

#### Risks Associated With Breach of Privacy

Patients voiced concerns about the implications of breaches of privacy. Common fears were discrimination, stigmatization, or psychosocial discomfort if medical information was shared with insurance companies or employers [22,23,51,63]. Similarly, employment and life insurance discrimination concerns were raised by community advisory group members regarding the presence of genomic information in the medical record [68]. In addition, concerns were expressed by some that genetic health information may affect their medical care—specifically the discontinuation or withholding of treatment [68]. In genetics research, patients also cited worries about release of information to family members without their consent [22,63]. Moreover, genetic information about risk for having a late-onset disease, such as Huntington’s, could lead to social stigmatization as well as insurance and employment discrimination [22,65,69].

#### Factors Related to Patients’ Views on Privacy

Three studies identified some individual factors related to views on privacy. Older people, described as individuals aged 50 years and older, reported less concern with privacy of genetic information than did younger people in a focus group study [58]. Gender differences were found related to the potential consequences of a security breach. Male patients reported concern about impacts on employment, finances, and insurance, whereas female patients were concerned about risks related to social discomfort and embarrassment [51]. Finally, those who had more experience with using computers were less concerned about security issues [59].

### Return of Research Results

#### Return of Individualized or Aggregate Results

Bioethicists have debated whether to return the results of genetic research to biobank participants. Much of the discussion has focused on the return of individualized versus aggregate results. Most agree that returning aggregate or summary results, through a newsletter or website, is appropriate. This is seen as both a measure of accountability for the researchers conducting the study and a sign of respect for the participants [23]. The provision of individual genetic results from a research study, though, has been a more contentious subject. Ethical arguments in favor of returning results are grounded in the principle of *respect for persons* and *beneficence* [23]. Having access to genetic information about oneself may have an impact on one’s health, quality of life, and future decision making*.* Returning individualized results to participants also prevents researchers from being gatekeepers of important information. However, those against sharing individual results cite that research and clinical care should not be blurred, and an investigator providing genetic results is acting more like a clinician than a researcher. In addition, those opposed state that research and clinical testing are quite different, with the goal of the latter being to provide better care to a patient, whereas the former is concerned with creating generalizable knowledge [23].

Given this debate, several ethics boards and research committees have created recommendations or guidelines for when to share individualized results, including for which genetic conditions [31,70,71]. The eMERGE network has also addressed this issue [72]. Among these guidelines, there is consensus that individual results should only be returned if they are medically actionable, even if they were incidental findings. Researchers are also encouraged to thoughtfully consider the language in the informed consent form that details what types of results will be shared with participants [22]. However, in a recent study that examined informed consent forms, the majority of consent forms did not discuss return of results [73]. The authors acknowledge, though, that many of these forms may have been created years before any guidance was offered on this issue.

#### Patient Preference for Return of Research Results

When patients have been asked about their preferences for the return of research results, most indicate they want to know. In a survey of patients enrolled in a Health Maintenance Organization (HMO) plan, about two-thirds said they would be willing to participate in research within the HMO if written information about the study was provided to them and almost three-fourths if results were provided [74]. Another study asked participants about their views regarding collection of tissue samples and genetic testing [49]. Return of test results was strongly supported by all participants, with the highest level of support among those who were concerned about having a health condition that may be genetic. In a survey of preferences for return of results using donated tissue samples, just under half (129/271, 47.6%) reported they always wanted to be given individual results, 25.1% (68/271) wanted results only after the researcher had assessed the risks and benefits of sharing the information, and 27.3% (74/271) wanted results if their doctor thought the information would inform decision making related to their care [75]. Respondents who thought genetic information was more sensitive than other types of health information were more likely to want to be told all individual results.

### Research on Vulnerable Populations

#### Genetic Research on Vulnerable Populations

Important ethical, legal, and social issues are raised when vulnerable populations are involved in genetic research [76]. Although similar to the issues already discussed, concerns related to recruitment, informed consent, privacy and security, and disclosure of results are often amplified when conducting research with vulnerable populations [23,77].

A clear example is when children are recruited to be part of genetic studies. One study asked adults whether they would want to be reconsented if their parents enrolled them in a biobank study when they were children. Overall, most respondents (799/1186, 67.37%) said they would be willing to continue participation, but almost half (543/1186, 45.78%) wanted to be reconsented [78]. In a review of procedures used in 6 different birth cohort studies, investigators found that blanket consent was never used. However, the studies varied in their approach to returning research results; although some provided participants and their families with routine clinical information and test results, other studies only informed participants if test results were abnormal for a treatable condition. Finally, all studies recontacted participants when they reached adulthood and allowed them the opportunity to withdraw or reconsent [79]. A recent study, though, examined an alternative consent model. When presented with an opt-out model of enrollment into a study that linked the child’s EHR with biospecimens, most parents were supportive [80]. Parents stated there was little risk given that the data would be deidentified. Although some were concerned about the security of EHR data, none raised reservations about access to genetic information.

#### Electronic Health Record Research on Vulnerable Populations

Although studies have been conducted using EHRs of vulnerable populations, such as epidemiological research [81-83], very few have examined the ethical, legal, and social issues related to this type of research. Simon and colleagues [84] discussed the risks and benefits of conducting research on individuals with psychiatric conditions using large medical databases. Although privacy risks are of critical concern, the authors argued that these population-based research methods have provided many benefits, including information on prevalence estimates, treatment effectiveness, and impact of health policy. With appropriate safeguards, this research can also lead to reducing stigmatization and discrimination through education and outreach efforts based on research findings.

## Discussion

### Summary of Findings

In this study, we sought to better understand the ethical, legal, and social issues related to the use of EHRs of individuals with intellectual disabilities by examining similar issues, as well as patients’ preferences, that have been raised in EHR research among the general public and in genetics or biospecimen research. The main themes we highlighted were related to obtaining informed consent, privacy and security of data, and the return of research results. With the push to expand precision medicine initiatives and EHR research to a diverse group of participants, it is important for researchers to consider the implications of these issues for individuals with genetic conditions, particularly those that result in intellectual disabilities. Below, we pose questions about each of these issues that researchers should consider when designing methods to involve those with intellectual disabilities in EHR research.

Given the issues related to informed consent that have been raised in prior studies, what are the best models of informed consent for use with individuals with genetic conditions who have intellectual disabilities? Is a broad or blanket consent model appropriate for this population? Would an opt-out model be ethically acceptable? or is the tiered consent model the only viable option? and would the type of consent model applicable for adults also be applicable for children with genetic conditions that result in intellectual disability? To answer these important questions, researchers should consider the decisional capacity of the individuals being asked to participate in the study. For example, individuals with fragile X syndrome, the most common inherited form of intellectual disability, may present with a range of impairment, from normal or mild delays to severe intellectual disability. Young children with a genetic condition, no matter their level of functioning, will require permission from a parent or guardian to participate in EHR research. However, when children with fragile X syndrome or other genetic conditions that result in intellectual disability reach adulthood, their capacity to consent should be assessed to determine if they adequately understand the study, including the risks and benefits of participating.

A large body of research has examined the capacity to consent in individuals with Alzheimer or other forms of cognitive impairment [85-88], and a handful have examined decisional capacity in individuals with intellectual disability [89-91]. This literature, combined with the ethical, legal, and social issues raised in this study, will help to inform the proper consent models for EHR researchers interested in recruiting participants who have a genetic condition that may result in intellectual disability. Experts agree, though, that a shared decision-making process should be employed to support these individuals to make informed consent choices [92-94].

Another set of questions arise regarding the privacy and security of EHR research conducted with individuals with intellectual disability that stems from a known or suspected genetic condition. Will these individuals have the same concerns as the general public regarding privacy and security of their EHR data, including who has access and what information researchers can see? Will these individuals be more reluctant to participate in EHR research due to the risk of identifiability, even if data have been deidentified? Or will they be less worried given that they may have more to benefit from EHR research? Much of the potential vulnerabilities of individuals with intellectual disabilities is related to the fear of coercion or possible stigmatization or discrimination that could result from misuse of research data [95].

EHR research conducted by pharmaceutical companies may trigger either support or apprehension from individuals with intellectual disability. Pharmaceutical companies play important roles in partnering with researchers to develop and test treatments; however, pharmaceutical companies may also be viewed unfavorably given the high cost of orphan drugs for some rare genetic conditions [95-98]. Similarly, those with an intellectual disability may be more or less willing to share sensitive or identifiable data with EHR researchers. If an individual has already been diagnosed with a genetic condition that results in intellectual disability, the risk of identification may be less significant if the individual’s diagnosis is commonly known. But, for those with very rare genetic diseases, there may be a heightened risk of identifiability, mainly because there are so few patients with a given condition [99]. It is difficult to answer these questions because few studies have examined the preferences of individuals with intellectual disabilities about participating in research more broadly [100], and none have asked about EHR research in particular. More work is needed to understand the implications of privacy and security issues for individuals with intellectual disabilities to help researchers determine how best to address them. At a minimum, the risks and benefits related to privacy and security should be carefully outlined in the informed consent form so individuals with intellectual disabilities and their parents or legally authorized representative can weigh the risks and benefits of participation and make informed decisions. Ideally, EHR research and informed consent forms will continue to evolve as laws and other privacy safeguards for the use of clinical data are updated [101].

The central question about the return of EHR research results for individuals with intellectual disabilities is whether individual or aggregate results should be provided to participants. The same potential for the blurring of the lines between clinical care and research applies here as it does for those in the general public who join biobanks as “healthy” individuals. If a secondary condition related to a genetic condition is discovered through EHR research, is it necessary to notify the participant? The answer may lie in what is contained in the informed consent form. If the secondary finding is medically actionable and participants agree to be informed, then results should be returned. But, if the result does not require immediate medical attention, then researchers may not necessarily need to disclose it. Researchers should carefully consider the types of information they may learn from EHR research involving individuals with intellectual disabilities and determine *a priori* what types of results would qualify as needing to be returned. Then, participants should be provided with a choice as to whether they wish to be notified. If individual results are not returned, then aggregate results in the form of patient-friendly summary reports or newsletters should be considered [102].

### Limitations and Future Research

It is important to note there were limitations in our approach. First, although we were broad in our search terms and criteria, we may have missed pertinent articles that should have been included. Similarly, because this was a scoping review and not a systematic review, there may be other ethical, legal, or social issues related to EHR research on individuals with intellectual disabilities that we do not cover. Finally, although we reviewed articles that included both US and non-US studies, our review of informed consent and federal laws were limited to those in the United States.

In conclusion, the issues highlighted in our review of the literature provide a framework for researchers to consider when conducting EHR research with individuals with intellectual disabilities. Future research should focus on further understanding the ethical, legal, and social issues in this type of research by asking individuals with intellectual disabilities, and their parents, for direct input on their preferences. Both qualitative and quantitative approaches will provide valuable information for researchers, so they can design studies that are more inclusive of this population using appropriate safeguards and protections, as needed.

## Abbreviations

EHR: electronic health record
eMERGE: Electronic Medical Records and Genomics
HIPAA: Health Insurance Portability and Accountability Act
HMO: Health Maintenance Organization
IRB: institutional review board
